# Ozone‐induced changes in the murine lung extracellular vesicle small RNA landscape

**DOI:** 10.14814/phy2.15054

**Published:** 2021-09-23

**Authors:** Gregory J. Smith, Adelaide Tovar, Matt Kanke, Yong Wang, Jessy S. Deshane, Praveen Sethupathy, Samir N. P. Kelada

**Affiliations:** ^1^ Department of Genetics University of North Carolina at Chapel Hill Chapel Hill North Carolina USA; ^2^ Department of Biomedical Sciences College of Veterinary Medicine Cornell University Ithaca New York USA; ^3^ Division of Pulmonary Allergy, and Critical Care Department of Medicine School of Medicine University of Alabama at Birmingham Birmingham Alabama USA

**Keywords:** air pollution, exosomes, extracellular vesicles, inflammation, lung, microRNA, mouse, ozone

## Abstract

Inhalation exposure to ozone (O_3_) causes adverse respiratory health effects that result from airway inflammation, a complex response mediated in part by changes to airway cellular transcriptional programs. These programs may be regulated by microRNAs transferred between cells (e.g., epithelial cells and macrophages) via extracellular vesicles (EV miRNA). To explore this, we exposed female C57BL/6J mice to filtered air (FA), 1, or 2 ppm O_3_ by inhalation and collected bronchoalveolar lavage fluid (BALF) 21 h later for markers of airway inflammation, EVs, and EV miRNA. Both concentrations of O_3_ significantly increased markers of inflammation (neutrophils), injury (total protein), and the number of EV‐sized particles in the BALF. Imagestream analysis indicated a substantial portion of particles was positive for canonical EV markers (CD81, CD51), and Siglec‐F, a marker of alveolar macrophages. Using high‐throughput small RNA sequencing, we identified several differentially expressed (DE) BALF EV miRNAs after 1 ppm (16 DE miRNAs) and 2 ppm (99 DE miRNAs) O_3_ versus FA exposure. O_3_ concentration‐response patterns in EV miRNA expression were apparent, particularly for miR‐2137, miR‐126‐3p, and miR‐351‐5p. Integrative analysis of EV miRNA expression and airway cellular mRNA expression identified EV miR‐22‐3p as a candidate regulator of transcriptomic responses to O_3_ in airway macrophages. In contrast, we did not identify candidate miRNA regulators of mRNA expression data from conducting airways (predominantly composed of epithelial cells). In summary, our data show that O_3_ exposure alters EV release and EV miRNA expression, suggesting that further investigation of EVs may provide insight into their effects on airway macrophage function and other mechanisms of O_3_‐induced respiratory inflammation.

## Graphical abstract

Respiratory exposure to the common ambient air pollutant, ozone, causes airway inflammation and injury. Using high‐throughput RNA sequencing, we investigated whether ozone‐induced inflammation was associated with altered lung extracellular vesicle small RNA expression. We found that specific extracellular vesicle miRNAs, including miR‐22‐3p, were altered by ozone exposure that may affect the function of airway cells.

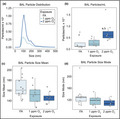

## 1 INTRODUCTION

Ozone (O_3_) is a highly reactive, oxidant air pollutant associated with significant adverse respiratory health effects including airway inflammation and exacerbation of respiratory diseases such as asthma (Al‐Hegelan et al., 2011). Observations of the health effects of O_3_ date to the mid‐19th century (Schönbein,), and yet, knowledge of the underlying mechanisms is still incomplete. Ground level O_3_ concentrations in many regions regularly exceed the current US national standard (0.07 ppm) (U.S. Environmental Protection Agency, 2020) and O_3_‐induced airway inflammation can occur at even lower concentrations (Kim et al., 2011). Given that ambient O_3_ concentrations are expected to rise in the coming decades due to climate change (Pfister et al., 2014), research to identify the mechanisms by which O_3_ causes respiratory health effects is still an imperative.

Inhaled O_3_ reacts rapidly with the airway surface lining liquid to generate reactive oxygen species, lipid peroxides, and other products that induce a complex and dynamic respiratory tract response (Mudway & Kelly, 2000; Pryor et al., 1995). Inflammation occurs up to 48 h following a single exposure, initiated in part, by the release of cytokines and marked by edema and the influx of neutrophils to the lung (Mudway & Kelly, 2000). Following the cessation of exposure, the anti‐inflammatory and pro‐resolving activities of the respiratory tract and innate immune cells restore homeostasis by about 72 h (Kilburg‐Basnyat et al.,).

Alveolar macrophages and epithelial cells are two airway cell types with readily apparent functional responses to O_3_ (Al‐Hegelan et al., 2011). Alveolar macrophages show reduced phagocytosis (Gilmour et al., 1991) and enhanced antigen presentation activity (Lay et al.,). Epithelial cell barrier dysfunction and damage are evidenced by the presence of an intra‐alveolar, albumin‐rich exudate following O_3_ exposure (Hollingsworth et al., 2007). Shared functional responses, albeit differing with respect to timing and precise mediators, include the release of pro‐inflammatory cytokines (e.g., IL‐6 (Devlin et al., 1991), IL‐1 family (Michaudel et al., 2016), TNFα (Fakhrzadeh et al., 2008)) and altered production/activity of other proteins (e.g., surfactant (Haque et al., 2007), metallothionein (Inoue et al., 2008), matrix metalloproteinases (Yoon et al., 2007)), and small molecules (e.g., antioxidants (Behndig et al., 2009), eicosanoids (Devlin et al., 1991), specialized pro‐resolving lipid mediators (Kilburg‐Basnyat et al.,)).

Underlying the functional cellular responses of airway macrophage and epithelial cells to O_3_ are alterations in inflammatory, immune, and oxidative stress response gene expression programs. Transcriptomic and proteomic studies have better illuminated the landscape of gene expression responses to O_3_, revealing the involvement of the protease/anti‐protease system (Kesic et al., 2012), heat shock proteins (Nadadur et al., 2005), NRF2 (Cho et al., 2013, 2013), and NF‐kB family transcription factors (Jaspers et al., 1997). Although the gene expression landscape of the airway response to O_3_ is becoming clearer, the precise mechanisms regulating gene expression responses to O_3_ are unknown.

Extracellular vesicles (EVs) have emerged as an important means of airway intercellular communication and may directly influence gene expression responses of the respiratory tract (Guiot et al., 2019; McVey et al., 2019; Raposo & Stoorvogel, 2013). EVs are a heterogeneous population of nanometer scale, lipid membrane‐bound particles, which includes exosomes and microvesicles (Raposo & Stoorvogel, 2013). EVs carry several classes of cargo including small RNAs such as microRNAs (miRNA) (Crescitelli et al., 2013). miRNAs regulate gene expression post‐transcriptionally by destabilizing or inhibiting the translation of target mRNAs, and a single miRNA can regulate hundreds of different mRNAs (Bartel, 2018). EV‐derived miRNAs (EV miRNAs) are readily detectable in the airway lumen and are taken up by respiratory tract cells including airway macrophages and epithelial cells (Guiot et al., 2019). As such, EVs represent a unique mechanism for the transfer of miRNA and intercellular miRNA‐mediated regulation of airway gene expression.

Inflammatory lung diseases including asthma (Levänen et al., 2013), idiopathic pulmonary fibrosis (Njock et al., 2018), and chronic obstructive pulmonary disease (COPD) (Fujita et al., 2015) as well as oxidant‐ or allergen‐induced lung inflammation (Lee et al., 2019; Pua et al., 2019) are associated with altered airway EV miRNA profiles. In addition, there is growing evidence that EV miRNAs directly alter airway cellular gene expression to change cellular phenotype. For example, studies have demonstrated their direct effects on myofibroblast and macrophage differentiation (Ismail et al., 2013; Saha et al., 2016) and protein secretion by epithelial cells (Gupta et al., 2019). Thus, it is likely that EV miRNA play functional roles in O_3_‐induced inflammation (Andres et al., 2020); however, the extent to which O_3_ exposure affects EV miRNA expression is unknown. We hypothesized that O_3_ exposure dysregulates airway EV miRNAs, thereby affecting target mRNA expression in cells of the airway including epithelial cells and macrophages. To investigate this, we conducted high‐throughput small RNA sequencing of miRNA isolated from murine airway EVs following filtered air (FA), 1. and 2 ppm O_3_ exposure and used an integrative bioinformatics approach to identify putative EV miRNA regulators of gene expression in airway macrophages and conducting airways. Our results provide a characterization of the murine airway EV miRNA landscape after ozone exposure, including specific EV miRNAs that may act as regulators of O_3_‐induced gene expression changes in the airway.

## 2 MATERIALS AND METHODS

### 2.1 Animals

Adult (Cho et al., 2013; Cobos Jiménez et al., 2014; Crescitelli et al., 2013) female C57BL/6J mice were purchased from the Jackson Laboratory and used for all experiments. We used female mice because prior data have shown greater inflammatory responses to O_3_ in this sex. Specifically, female mice display increased pro‐inflammatory gene expression profiles, secretion of cytokines and chemokines, and neutrophil influx in response to acute O_3_ exposure (Cabello et al., 2015; Mishra et al., 2016), thus focusing on this sex could provide an opportunity to detect a more dynamic transcriptional response. Mice were housed in cages at a maximum density of five per cage on ALPHA‐Dri bedding (Shepard) under standard 12 h lighting conditions, with ad libitum access to food (Envigo 2929) and water. After a 1‐week acclimation period, mice were randomly assigned to different exposure groups. The sample size for each group is indicated in the figure legends. All experiments were conducted within an AAALAC approved facility and were approved by the Institutional Animal Care and Use Committee (IACUC) at the University of North Carolina at Chapel Hill.

### 2.2 Ozone exposures

Whole body inhalation exposures to filtered air (FA), 1, or 2 ppm O_3_ for 3 h were conducted as previously described (Smith et al., 2019; Tovar et al., 2020). Environmental conditions for each experiment, including mean O_3_ concentration (Supplemental Figure S1), temperature, and relative humidity were recorded (Supplemental Table S1).

### 2.3 Bronchoalveolar lavage

Mice were euthanized 21 h following cessation of exposure with a lethal dose of urethane (2 g/kg, i.p.) followed by exsanguination. We chose a 21‐h time point based on our previous work (Tovar et al., 2020) which showed abundant transcriptional responses to ozone at this time point.

Bronchoalveolar lavage was conducted with a 21‐gauge catheter inserted in the trachea. The lung was then gently lavaged twice (1 × 0.5 ml, 1 × 1 ml) with ice‐cold PBS containing complete protease inhibitor cocktail (Roche) using a 1‐ml syringe. Collected bronchoalveolar lavage fluid (BALF) was centrifuged (400 x g, 10 mins, 4ºC) to pellet cells. Red blood cells were lysed with hypertonic saline and the remaining white blood cells were resuspended in Hank's Balanced Salt Solution and counted by hemocytometer and mounted on slides via cytospin. Cytospin slides were stained with Kwik‐Diff (Shandon). Two investigators, blinded to the exposure group, performed differential cell counts (minimum 300 cells per slide).

### 2.4 EV isolation and characterization

After the removal of cells from BALF, a second centrifugation was performed (16,000 x g, 10 mins, 4ºC) to pellet cellular debris and larger vesicles (e.g., apoptotic bodies). The concentration and size distribution of particles within the BALF supernatants were measured using the Nanosight NS500 Nanoparticle Tracking Analysis system (Malvern Panalytical) at the UNC Nanomedicines Characterization Core Facility. BALF supernatant samples were diluted 1:100 in 0.02‐μM filtered PBS prior to loading on the NTA instrument. This level of dilution provided visualization of between 10 and 50 particles/frame. Five individual, 40‐s long, motion captures of the suspended particles were analyzed using Nanosight software v3.2 (Malvern Panalytical). Specific camera settings included camera levels of 14–16 and detection thresholds of 4–5. Nanoparticle Tracking Analysis instrument calibration was performed using 100 nm polystyrene beads (Malvern Panalytical).

### 2.5 ImageStream analysis of EVs

Intact EVs were isolated from BALF using an ExoRNeasy column purification and eluted in 400 µl of buffer XE (Qiagen). ImageStream analysis was performed on eluted EVs (Hough et al., 2018). EVs were stained with the following antibodies: FITC‐conjugated anti‐mouse CD326 (EpCAM, clone G8.8, Life Technologies). PE‐conjugated anti‐mouse CD81 (clone Eat‐2), PE‐Cy7‐conjugated anti‐mouse CD9 (clone MZ3), PE‐Cy7‐conjugated anti‐mouse CD31 (clone 390), Alexa 594‐conjugated anti‐mouse CD11b (clone M1/70), BV421‐conjugated anti‐mouse CD170 (Siglec‐F, clone S17007L), APC‐conjugated anti‐mouse CD63 (clone NVG‐2) and APC‐conjugated anti‐mouse CD170 (Siglec‐F, clone S17007L) purchased from BioLegend. FITC‐conjugated anti‐mouse Ly6G (clone 1A8) and BV421‐conjugated anti‐mouse CD51 (clone RMV‐7) purchased from BD Biosciences. Unstained control, single color staining, and calibration beads were used to calibrate the machine and adjust compensation. The stained samples were imaged at 60X magnification without extended depth of field. The data were acquired on channels Ch01, Ch02, Ch03, Ch04, Ch06, Ch07, Ch09, Ch11, and Ch12. Ch01 and Ch09 were used as bright field channels, whereas Ch12 was used for side‐scatter. A total of 5,000 events were acquired, gating on forward‐ and side‐scatters, as well as aspect ratio and area. The acquired data were analyzed using IDEAS software version 6.2 (EMD Millipore).

### 2.6 EV small RNA isolation and sequencing

BALF samples were pooled into groups of three to four and total RNA was isolated from pooled samples using an ExoRNeasy Serum/Plasma Midi kit (Qiagen). Column‐bound EVs were lysed with Qiazol and total EV RNA was isolated using a Qiagen RNeasy MinElute spin column (Qiagen). Prior to sequencing, the yield and integrity of small RNA from isolated and lysed EVs were measured using an Agilent 2100 Bioanalyzer with small RNA chip, and an example result is shown in Supplementary Figure S4B. Small RNA sequencing libraries were prepared using a BioO Scientific NEXT Flex‐v3 kit. Single end sequencing was performed on the Illumina HiSeq 4000 platform at the UNC High‐Throughput Sequencing Facility (University of North Carolina, Chapel Hill, NC, USA).

### 2.7 EV small RNA expression analysis

Using miRquant 2.0 (Kanke et al., 2016), small RNA reads were trimmed of adapters, aligned to the *Mus musculus domesticus* reference genome (assembly NCBI37/mm9), and miRNAs and isomiRs (miRNA sequence variants) were quantified. Small RNA Bioanalyzer results, read length and mapping statistics are presented in the results section and Supplemental Figures S4 and S5.

In follow‐up analyses, we performed qRT‐PCR to quantify miR‐22 levels. cDNA synthesis was performed on 10 ng of EV‐derived RNA using a TaqMan advanced miRNA cDNA Synthesis kit (ThermoFisher Scientific). miRNA qPCR reactions were performed in triplicate on a BioRad CFX 384 Touch Real‐Time PCR detection system using TaqMan Fast Advanced qPCR Master Mix (ThermoFisher Scientific) according to the manufacturer's instructions. TaqMan Advanced miRNA assays for miR‐22‐3p (mmu481004_mir) and miR‐23a‐3p (mmu478532_mir) were used for RT‐qPCR expression analysis. miR‐23a‐3p was used for normalization based on its low coefficient of variation in expression across samples measured by small RNA sequencing. miRNA levels are expressed as relative quantitative values (RQVs) and statistical analyses were conducted using dCq values.

### 2.8 EV miRNA target mRNA prediction

miRNA target site enrichment analysis was performed on lists of O_3_‐responsive genes from our previously published (Tovar et al., 2020) RNA sequencing analysis of airway macrophages (isolated from BALF) and conducting airway tissues (microdissected from lungs preserved in RNAlater (ThermoFisher Scientific) using the bioinformatics algorithm mirRhub (Baran‐Gale et al., 2013). Using a list of up‐ or downregulated genes as an input, miRhub was used to determine if any miRNA target sites were significantly over represented in these lists of previously identified O_3_‐responsive genes (Tovar et al., 2020) compared to randomly generated gene lists. Only miRNAs that were conserved in two other species and whose expression was directionally opposed to the tissue mRNA patterns across the exposure groups were considered. For a designated contrast, both the miRNA and mRNA were required to be significantly differentially expressed (fold change of ±2, *p*‐adjusted <0.05).

### 2.9 Statistical analysis

**FIGURE 1:**
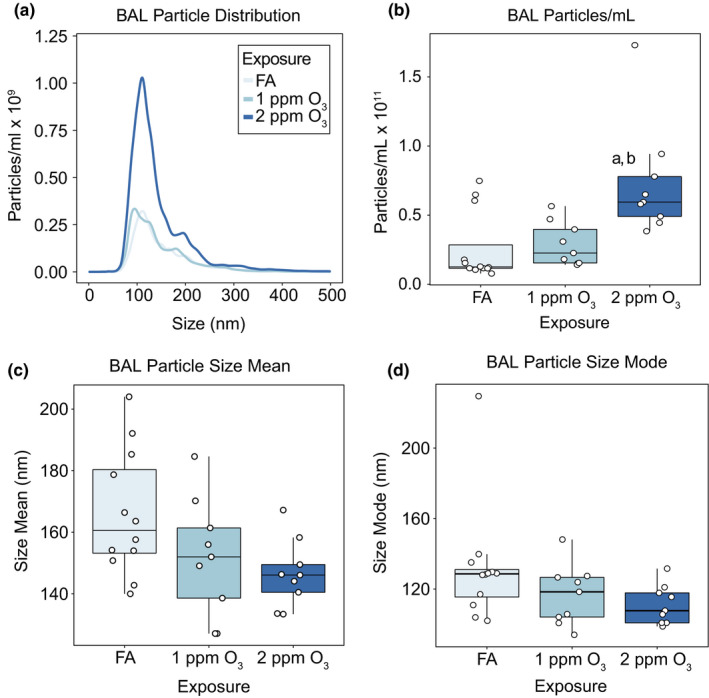
O_3_ exposure causes increases in EV‐sized particles in the lung. Female C57BL/6J mice were exposed to filtered air (FA; n = 12), 1 (n = 9), or 2 (n = 9) ppm O_3_ for 3 hours and bronchoalveolar lavage fluid was collected 21 hours later for isolation and analysis of extracellular vesicles. (A) Size distribution, (B) total concentration, (C) size mean, and (D) size mode of particles in BALF samples were measured by nanoparticle tracking analysis. Results are depicted in (A) as the mean particle‐size distribution. In (B‐D) Box‐and‐Whisker plots depict the minimum, first quartile, median, third quartile, and maximum of the data with all points overlaid. a: *p* < 0.05 compared to the FA group, b: *p* < 0.05 compared to the 1 ppm group. In (C) and (D), linear regression models demonstrated significant relationship between O_3_ concentration and size, mean and mode, p‐value =0.016 and 0.049, respectively

**FIGURE 2:**
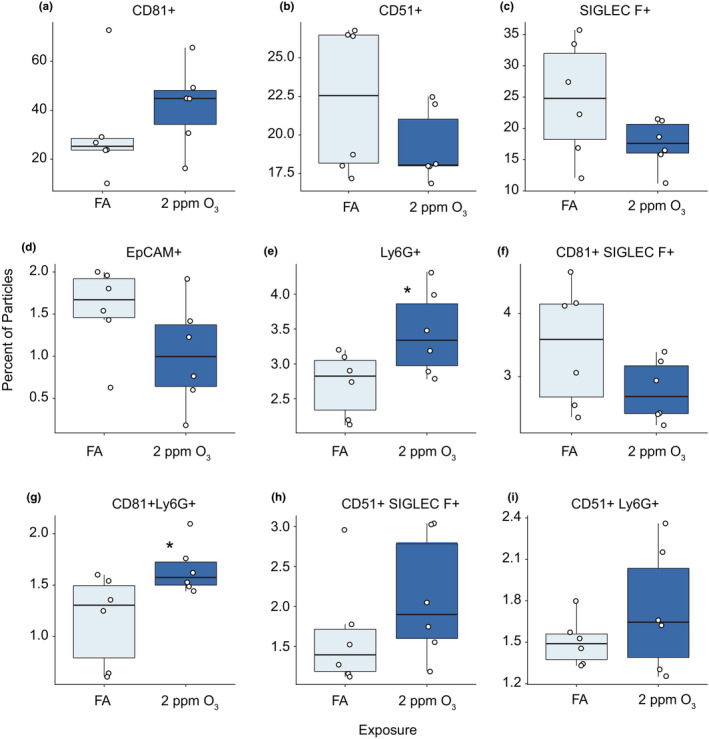
Imaging flow cytometry analysis of BALF EVs. Percentage of EVs positive for (A) CD81, (B) CD51, (C) Siglec‐F, (D) EpCAM, (E) Ly6G, (F) CD81 and Siglec‐F, (G) CD81 and Ly6G, (H) CD51 and Siglec‐F, and (I) CD51 and Ly6G. EVs were isolated for ImageStream analysis from mice 21 hours following a 3‐hour exposure to FA or 2 ppm O_3_. n = 6 per group. Need to add some indication of stat test results here

Statistical analyses were conducted using R (version 3.6.1). For analysis presented in Figure 1b, and Supplemental Figure S2 a Box‐Cox procedure was used to determine an appropriate transformation of the data to reduce heteroscedasticity and conform to a normal distribution prior to ANOVA and Tukey's HSD post hoc tests for group comparisons. In Figure 1c, d, linear regression models were used to determine whether any statistically significant concentration–response trends were present. *T*‐tests were used for Figure 2 to compare 2 ppm O_3_ to the FA group.

## 3 RESULTS

### 3.1 Ozone (O_3_) inhalation causes inflammation and increased extracellular vesicle (EV)‐sized particles in the lung

We exposed female C57BL6/J mice to filtered air (FA), 1, or 2 ppm O_3_ for 3 h and collected bronchoalveolar lavage fluid (BALF) 21 h later for isolation of EVs and measurement of lung inflammation and injury. As previously reported (Tovar et al., 2020), BALF samples from both 1‐ and 2‐ppm O_3_‐exposed mice exhibited characteristic pulmonary inflammatory phenotypes including significant increases in cellularity, driven primarily by neutrophils, and injury as measured by increased total protein compared to FA (control) mice (Supplemental Figure S2). Both tissue injury and inflammation were most severe in the 2‐ppm O_3_‐exposed group compared to the 1‐ppm O_3_ group.

We measured the number of particles in BALF supernatants across a size range of ~50–1000 nm by nanoparticle tracking analysis. We observed a concentration‐dependent increase in the total number of particles in the size range of EVs, roughly 50–400 nm (Figure 1a). The increase in BALF particle concentration was statistically significant at 2 ppm O_3_ compared to the 1‐ppm O_3_ and FA groups (Figure 1b). Additionally, we observed modest shifts in the particle size distributions across treatment groups, with both the means and modes of particle size indicating a statistically significant decreasing trend from FA to 2 ppm O_3_ (Figure 1c, d). In an independent experiment, we compared the particle distributions in BALF supernatants (as in Figure 1a, b) with the distribution of BALF EVs isolated by membrane affinity column separation from aliquots of the same BAL supernatant samples (Supplemental Figure S3). Although column purification reduced the overall particle number, an increase in particle number remained evident in post‐column EVs from mice exposed to 2 ppm O_3_ compared to FA.

### 3.2 EV characterization by imaging flow cytometry

To assess the relative proportions of exosomes and microvesicles in BALF EV samples and provide estimates of their cellular source(s), we isolated and eluted intact BALF EVs for imaging flow cytometry. We observed a high proportion of BALF EVs expressing typical markers of exosomes (~25–40%, CD81) and microvesicles (~20%, CD51), respectively (Figure 2). Expression of other markers indicative of cell‐type origin, such as Siglec‐F (alveolar macrophages), EpCAM (epithelial cells), CD11b (non‐resident monocytes/macrophages), and Ly6G (neutrophils), each comprised a small proportion of the total EV particles (Figure 2 and Supplemental Table S2). There were more Ly6G‐positive EVs and exosomes (CD81^+^, Ly6G^+^) in 2‐ppm O_3_‐exposed mice (Figure 2 and Supplemental Table S2). We did not detect statistically significant effects of O_3_ exposure on the expression of the other EV or cell‐type markers, although an increase in exosomes (EVs expressing CD81) was noted (Figure 2a).

### 3.3 Ozone (O_3_) induces miRNA expression changes in lung EVs

To obtain sufficient quantities of small RNA for downstream sequencing, we pooled BALF from three to four mice per treatment group, isolated EV‐total RNA from each of the pools, and characterized RNA abundance and size distributions. RNA abundance did not differ between groups (Supplemental Figure S4a), and RNA contents from each pool were predominantly in the size range of 20–40 nucleotides (Supplemental Figure S4b). To identify O_3_‐responsive EV miRNAs, we performed high‐throughput small RNA sequencing (small RNA‐seq) on each of the pooled EV RNA samples (n = 3 pools/exposure group). We obtained 44 million reads per sample on average, 78% of which were unambiguously mapped to the reference mouse genome (Supplemental Figure S5a). The bulk of mapped reads (~70–90%) were in the range of 30–35 nucleotides and mapped to tRNA, suggestive of tRNA‐derived RNA fragments (tDRs) (Supplemental Figure S5b and c). miRNAs constituted 1–2% of mapped reads, and the balance consisted of other small, unannotated RNAs (e.g., yRNAs, partially degraded longer RNAs.). Across exposure groups, we observed an increase in the percentage of reads mapped to these other small RNAs and a decrease in reads mapped to tRNAs, while the percentage of miRNA was reduced by half in the 2‐ppm O_3_ exposure group from ~2 to ~1%. Despite their relatively low abundance, we focused on EV miRNAs because of their known roles in modifying gene expression in target cells (Bartel, 2018; Raposo & Stoorvogel, 2013; Zhang et al., 2019).

**FIGURE 3:**
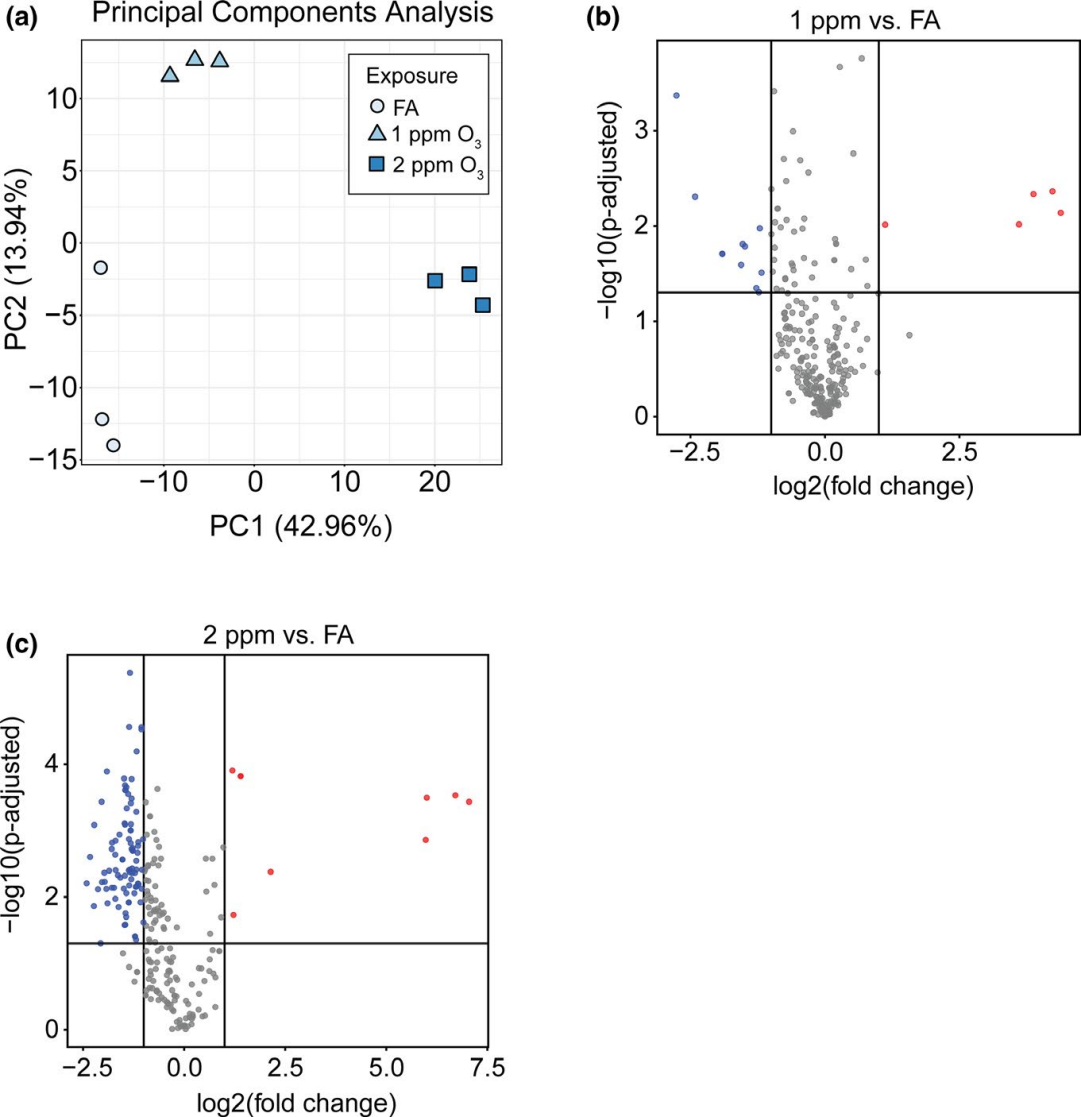
O_3_ exposure alters airway extracellular vesicle miRNA expression. (A) Principal components analysis showing separation of pooled airway EV samples by exposure group. (B and C) Volcano plots showing differentially expressed (DE) miRNAs in 1 ppm O_3_ versus FA (16 DE miRNAs) and 2 ppm O_3_ versus FA (99 DE miRNAs), respectively (horizontal line: p ‐adjusted = 0.05, vertical lines: absolute fold change of ±2). N = 3 EV RNA samples per exposure group, where each sample is a pool of 3–4 individual mice

**FIGURE 4:**
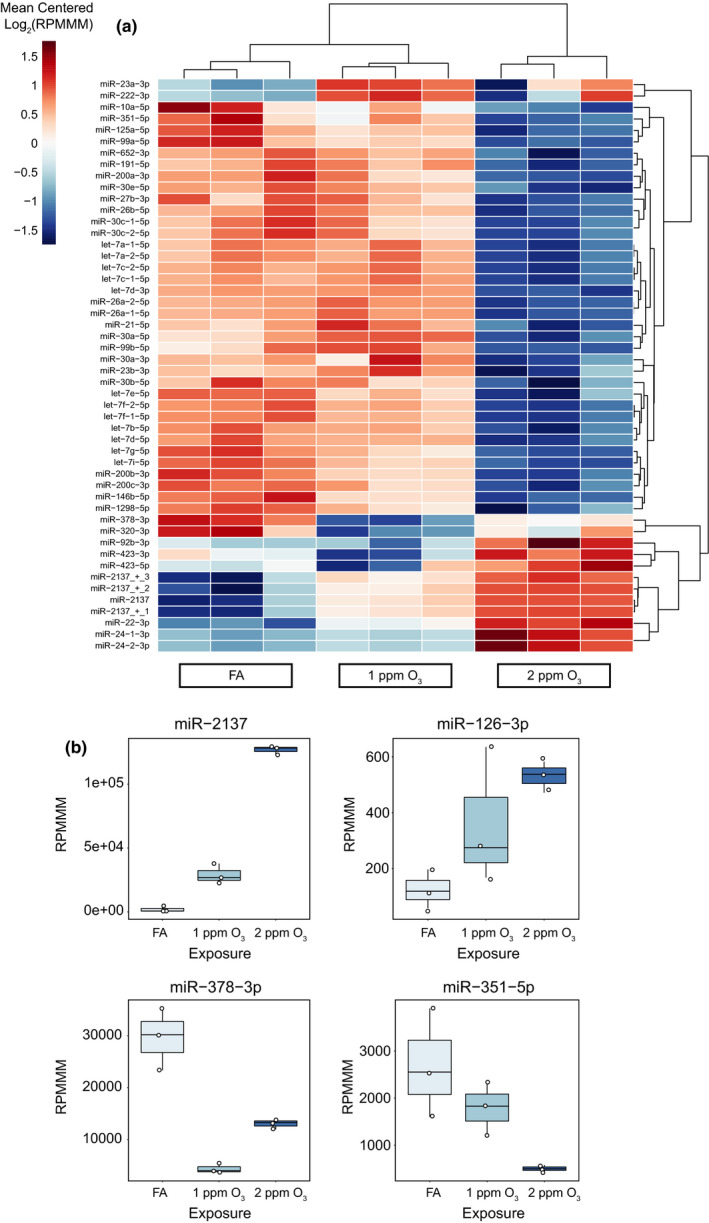
Specific O_3_‐induced alterations of the EV miRNA landscape. (A) Unsupervised hierarchical clustering analysis of the top 50 most variable EV miRNAs. Data are presented as mean‐centered, log_2_‐transformed reads per million miRNAs mapped (RPMMM) for each miRNA. (B) Expression of selected O_3_‐responsive EV miRNAs. Box‐and‐Whisker plots depict RPMMM summarized by the minimum, first quartile, median, third quartile, and maximum with all points overlaid. n = 3 pooled samples per group

We performed principal component analysis (PCA) on the 50 most variably expressed EV miRNAs, which revealed distinct EV miRNA expression patterns by exposure (Figure 3a). Using a threshold of fold change of ±2, *p*‐adjusted <0.05, and expression level of >50 reads per million miRNAs mapped (RPMMM), we identified 16 significantly differentially expressed EV miRNAs after 1 ppm O_3_ exposure and 99 significantly differentially expressed EV miRNAs after 2 ppm O_3_ exposure (Figure 3b, c). Overall, we observed a greater proportion of downregulated versus upregulated EV miRNAs in both the 1‐ and 2‐ppm O_3_‐exposed groups compared to FA (Figure 3b and c). Hierarchical clustering of the 50 most variably expressed EV miRNAs revealed several different concentration‐response patterns of expression including monotonic and non‐monotonic relationships (Figure 4a), as we previously observed for mRNAs (Tovar et al., 2020). An apparent threshold effect was observed for the bulk of EV miRNAs, illustrated by the closer correlation of the FA and 1‐ppm O_3_ groups compared to the 2‐ppm O_3_ group (Figure 4a). The most highly upregulated EV miRNA after both 1 and 2 ppm O_3_ exposure was miR‐2137 (Figure 4b) as well as several miR‐2137 isomiRs generated by alternative processing or nucleotide additions (Supplemental Figure S6). In addition to miR‐2137, miR‐126‐3p was one of the few upregulated miRNAs overall (~3.0‐ and 4.4‐fold in 1 and 2 ppm O_3_, respectively, vs. FA) (Figure 4b). The most highly downregulated miRNAs were miR‐378‐3p (~6.8‐ and 2.3‐fold, 1 and 2 ppm O_3_ vs. FA) and miR‐351‐5p (~1.5‐ and 5.3‐fold, 1 and 2 ppm O_3_, respectively, vs. FA) (Figure 4b).

### 3.4 Integration of EV miRNA and tissue mRNA data to identify putative regulatory relationships

Previously, we characterized the transcriptional profiles of airway macrophages and conducting airway tissue of mice exposed to O_3_ (Tovar et al., 2020). We integrated those mRNA expression data with the EV miRNA data presented here as a strategy to identify EV miRNAs that may cause the observed changes in tissue mRNA expression. Specifically, we sought to identify EV miRNAs that could plausibly regulate sets of target mRNAs in recipient airway macrophage or conducting airway tissue. Given the canonical role of miRNAs in decreasing mRNA levels and/or inhibiting mRNA translation (Bartel, 2018), we focused our analysis on EV miRNAs whose expression was directionally opposed to the tissue mRNA patterns across the exposure groups (i.e., we paired miRNAs that increased after 1 and 2 ppm O_3_ with mRNAs that correspondingly decreased, or vice versa), and we stipulated that both miRNA and mRNA must be significantly differentially expressed for the designated contrast.

**FIGURE 5:**
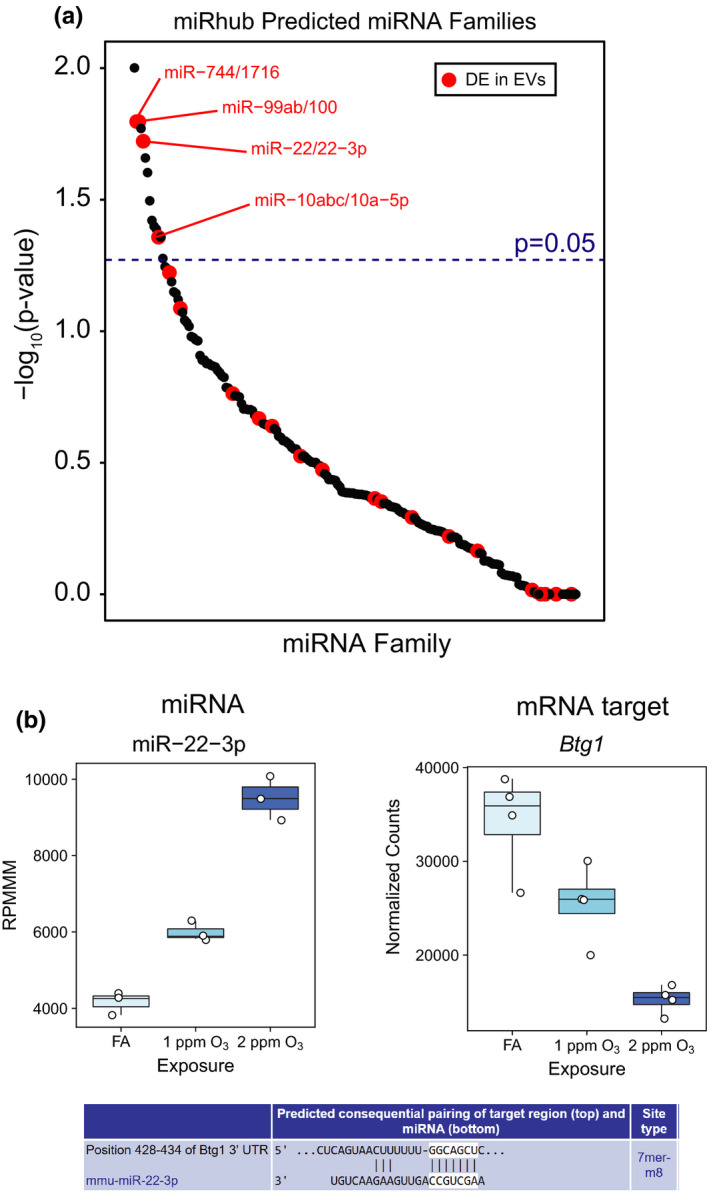
Identification of candidate EV miRNA regulators of O_3_‐induced transcriptional responses. (A) Results of miRNA target site enrichment analysis, conducted using miRhub, on genes downregulated by 2 ppm O_3_ versus FA in airway macrophages. Data points are plotted as –log_10_(*p*‐value) versus miRNA family and are highlighted if a member of the miRNA family was found to be differentially expressed in airway EVs. (B) Plot depicting RPMMM for EV miR‐22‐3p alongside expression of a predicted airway macrophage mRNA target, *Btg1* (normalized counts from DESeq2), and the miR‐22‐3p target site in *Btg1* from TargetScan mouse release 7.2. n = 3 or 4 (mRNA data) pooled samples per group

Our analysis of regulatory relationships using miRhub identified several miRNAs that were predicted to target sets of genes in airway macrophages or conducting airways (the full set of results are shown in Supplemental Figure S7). EV miR‐22‐3p emerged as a particularly interesting candidate regulator of gene expression in airway macrophages (Figure 5a) because it was highly expressed (>1,000 RPMMM) and was predicted to target seven downregulated genes (*Kdm6b*, *Stk39*, *Btg1*, *N4bp2*, *Sp1*, *Dpysl3*, and *Cnot6l*). The pattern of O_3_‐induced expression of EV miR‐22‐3p is shown alongside expression in airway macrophages of one of its predicted target genes, *Btg1* (Figure 5b). Finally, we validated the effect of 2 ppm O_3_ on EV miR‐22‐3p expression in an independent experiment (Supplemental Figure S8).

## 4 DISCUSSION

In this study, we examined the effects of exposure to the common air pollutant O_3_ on the murine lung EV landscape and focused in particular on changes in EV small RNA content. We observed an increase in the number of EVs in the lungs in parallel with markers of inflammation (macrophages, neutrophils) and injury (total protein) following O_3_ exposure. O_3_ exposure also changed the profile of lung EV small RNAs, with 16 and 99 miRNAs up‐ and downregulated by 1 and 2 ppm O_3_, respectively. Our data suggest that these biologically active EV cargo are O_3_‐responsive, and as such, may play a role in regulating O_3_‐induced inflammation. We note that because we chose to use female mice, based on evidence of greater airway neutrophilia and inflammatory cytokine production in this sex (Cabello et al., 2015; Mishra et al., 2016), the sex‐dependence of our results is not known and thus should be addressed in future studies.

We isolated EVs from BALF, which, prior to processing represents a complex mixture of small molecules, lavageable cells, larger debris, and nanoparticles (40–1000 nm). These nanoparticles include both the lipid membrane‐bound EVs of interest and non‐EV particles such as protein‐aggregates. To process our BALF samples for EV isolation, we performed a centrifugation at 16,000 x g to remove apoptotic bodies and large debris, which resulted in depletion of particles greater than 400 nm. In these samples, we observed an increase in particles due to O_3_ exposure that was most evident in the size range of ~80–150 nm size range and a decrease in the size mode from ~120 to 100 nm, suggestive of exosome and small microvesicles (Raposo & Stoorvogel, 2013). Although these data only provide a rough estimate of the particle type and it is not possible to rule‐out a contribution of non‐EV particles, we confirmed that the O_3_‐induced increase in the number of particles was present after EVs were isolated from the BALF using membrane affinity column isolation.

Our characterization of surface marker expression in eluted EVs further demonstrated that the BALF EVs we isolated were composed primarily of exosomes and microvesicles, identified by typical exosome (CD81, CD9, and CD63) and microvesicle markers (CD51 and CD31) (Théry et al., 2019). EVs positive for the exosome marker CD81 predominated and appear to be more responsive to O_3_. Lung EVs derive from multiple cell types, and the largest proportion of EVs were Siglec‐F positive, suggestive of an alveolar macrophage origin. However, Siglec‐F‐positive EVs (CD81 and Siglec‐F) comprised only ~1.5–4% of the total population of EVs. We also observed a small set of CD11b‐positive EVs, indicative of monocytes/macrophage origin. Somewhat surprisingly, we identified few particles of epithelial cell origin, as evidenced by less than one percent EpCAM‐positive EVs in both the FA and O_3_ groups. Previous studies, using standard flow cytometry and different markers, identified greater proportions of epithelial cell‐ derived EVs in BALF samples collected from controls and following exposure to other inflammatory stimuli (Lee et al., 2018; Moon et al., 2015). Overall, differences in cell‐type origin were less dynamic in our study; other than a small but statistically significant increase in CD81‐ and Ly6G‐positive particles (indicative of neutrophil derived EVs), O_3_‐induced effects on surface marker expression were not observed. However, we do note that an important role for neutrophil‐derived EVs has been observed in a mouse model of COPD (Genschmer et al., 2019).

Our global genomic approach allowed us to identify changes in miRNA content of EVs derived from multiple cell types, but the question of which EV‐derived miRNAs come from which cell type remains challenging. In general, there were few O_3_‐induced changes in EV marker expression, suggesting that changes in cellular sources are not likely to explain the bulk of O_3_‐induced EV small miRNA expression changes (or other small RNA species). Future studies to delineate the regional and cellular sources of specific EV‐derived small RNA using flow‐sorting and/or fluorescence microscopy and microdissection based approaches would be worthwhile. Nevertheless, by using a global EV collection and small RNA‐seq approach, we identified a host of differentially expressed airway EV miRNAs following O_3_ exposure and specific miRNAs that may regulate transcriptional responses to O_3_ in the lung.

The overall number of differentially expressed EV miRNAs and magnitude of change in their expression was concentration‐dependent, with more differentially expressed EV miRNAs in the 2‐ versus 1‐ppm O_3_ group. Additionally, the majority of differentially expressed miRNA was downregulated, which may reflect either a selective decrease in EV loading or a loss of the miRNA’s cellular source due to toxicity. miR‐2137 was identified as the most highly upregulated and expressed EV miRNA across both the 1 (~14‐fold) and 2 ppm O_3_ (~120‐fold) groups. Recently, differential expression of miR‐2137 was observed in bone marrow‐derived macrophages after infection with *Porphyromonas gingivalis* bacteria (Huck et al., 2017), and inhibiting miR‐2137 increased expression of the anti‐inflammatory cytokine IL‐10, suggesting that miR‐2137 may be pro‐inflammatory following O_3_ exposure. We also observed an O_3_‐induced upregulation of EV miR‐126‐3p, a well‐studied pro‐inflammatory miRNA (Fogel et al., 2019). miR‐126‐3p is upregulated in inflammatory bowel disease (IBD), in which it targets nuclear factor‐kappaB inhibitor alpha (IκB) (Feng et al., 2012; Wu et al., 2008) and impairs intestinal epithelial barrier function (Chen et al., 2017). We observed a downregulation of miR‐378‐3p, whose overexpression has been shown to negatively regulate macrophage proliferation (Rückerl et al., 2012). Therefore, the known O_3_‐induced increase in macrophage proliferation is consistent with our results and a potential regulatory effect of EV miR‐378‐3p (Hotchkiss et al., 1989). miR‐351 was also downregulated, and recent evidence suggests that downregulation of miR‐351‐5p is anti‐inflammatory in ischemia/reperfusion injury (Zheng et al., 2019). As such, miR‐351‐5p may represent a pro‐resolving signal after O_3_ exposure. In general, our data suggest that lung EVs collected 21 h following O_3_ exposure contain a mixture of both pro‐inflammatory and pro‐resolving signals.

Our integrative bioinformatics analysis of EV miRNA expression identified miR‐22‐3p as a putative regulator of airway macrophage gene expression following O_3_ exposure. A validated target (Huang et al., 2020) of miR‐22‐3p, *Btg1*, exhibited a concentration‐response pattern of expression in airway macrophages consistent with miR‐22‐3p regulation. The product of *Btg1*, B‐cell translocation gene 1 (BTG1), is known to suppress cell growth and proliferation (Yuniati et al., 2019). Interestingly, miR‐22‐3p is differentially expressed across the continuum of polarized human macrophages (Cobos Jiménez et al., 2014) and can promote M2 polarization of macrophages by suppressing interferon regulatory factor 5 (IRF5) (Fang et al., 2021). These data suggest miR‐22‐3p may regulate anti‐inflammatory activities of airway macrophages following O_3_ exposure (Sunil et al., 2012). Surprisingly, although we identified putative miRNA regulators for genes expressed in conducting airway tissue, none of these miRNAs were differentially expressed in EVs due to O_3_ exposure. This could be due to timing; for example, an upregulated EV miRNA could have influenced gene expression in conducting airway tissue at a different time point and was no longer or not yet differentially expressed in EVs at 21 h. This may explain why miR‐2137 was not a predicted regulatory hub in either tissue despite its high expression in EVs. Overall, our results suggest that changes in EV miRNAs have a larger effect on gene expression in macrophages versus conducting airways at this time point.

In addition to quantifying specific miRNA expression, our small RNA sequencing approach also revealed the overall proportions of multiple small RNA species present in BALF EVs, including tRNAs, piRNA, and yRNA. We found the majority of BALF EV small RNA reads, from 60 to 90% across exposure groups, mapped to tRNAs. A recent study reported a high percentage of tRNA‐mapped reads in rodent serum EVs (Zhao et al., 2020). The tRNA content of our samples is consistent with tRNA‐derived RNA fragments (tDRs) based on read length. The biology of tDRs is not well understood; however, evidence suggests they may function similarly to miRNAs by regulating gene expression through RNA interference among other suggested functions (Soares & Santos, 2017). Thus, the precise annotation and quantification of O_3_‐induced murine EV tDRs may reveal additional biological insights into ozone's mechanism of action.

In conclusion, we show that the release of airway EVs and dysregulation of EV miRNA content are features of respiratory tract response to O_3_ exposure. In addition to O_3_‐induced increases in the number of EVs and EV miRNA content, O_3_ also induced broad changes in the quantity of other EV small RNA species such as tRNAs. Our findings contribute to the growing body of evidence that EV miRNAs are both altered by and regulate inflammation in a range of mucosal tissues, including airway responses to O_3_ and provide compelling support for a range of mechanistic follow‐up studies. In particular, addressing the question of whether changes in EV number and/or content, including miR‐22‐3p and miR‐2137 specifically, are responsible for changes in gene expression in macrophages and other cell types and consequently aspects of O_3_‐induced airway inflammation or the resolution thereof.

## AUTHOR CONTRIBUTIONS

G.J.S. conceived and designed the studies with guidance from S.N.P.K and P.S.; G.J.S. and A.T. performed the experiments; G.J.S and M.K. analyzed the data; J.S.D. and Y.W. conducted the imaging flow cytometry analyses, G.J.S and S.N.P.K. prepared the manuscript; all authors approved the final version of the manuscript.

## Supporting information



Fig S1‐S8

Supplementary Material

## Data Availability

All sequencing data have been uploaded to the NCBI Gene Expression Omnibus (GEO) under accession number: GSE181645. All phenotype data are included in the Supplemental Data Tables S1 and S2.
