# Supplementary material for: Ozone‐induced changes in the murine lung extracellular vesicle small RNA landscape
Source: Physiol Rep. 2021 Sep 23;9(18):e15054. doi: 10.14814/phy2.15054 (PMC8461034; doi:10.14814/phy2.15054)

Supplemental Figure Legends

Supplemental Figure 1. O_3 exposure concentration-time profiles. Data are presented as 30-second running averages of the O_3 concentration (ppm) for both the 1 ppm (blue line) and 2 ppm (red line) O_3 exposures. Horizontal dashed lines represent ± 3% of the nominal exposure concentration.

Supplemental Figure 2. O_3 exposure causes airway inflammation and injury. Nine to ten-week-old female C57BL/6J mice were exposed to filtered air (FA; n = 12), 1 (n = 9) or 2 (n = 9) ppm O_3 for 3 hours and bronchoalveolar lavage fluid (BALF) was collected at 21 hours for differential cell counting and total protein analysis. (A) Total number of cells, (B) percent neutrophils, (C) number of neutrophils, and (D) total protein in BALF samples. a: $p < 0.05$ compared to FA group, b: $p < 0.05$ compared to 1ppm group.

Supplemental Figure 3. O_3 exposure causes an increase in isolated lung EVs. Nine to ten-week-old female C57BL/6J mice were exposed to filtered air (FA) or 2 ppm O_3 for 3 hours and bronchoalveolar lavage fluid (BALF) was collected at 21 hours for isolation of EVs. To recapitulate the preparation method used for EV small RNAseq, one pool of BALF from 3 mice (3 mL total) per group were applied to affinity-columns and intact EVs were collected in 400 µL of elution buffer. Plots depict the distribution of (A) the particle concentration and (B) the number of particles normalized to sample volume (particle yield) for the BALF supernatant (pre-column) and eluted BALF EVs (post-column).

Supplemental Figure 4. Airway EV small RNA characteristics. RNA was isolated from pooled airway EV samples from mice exposed to filtered air (FA; 3 pools from 4 mice each), 1 (n = 3 pools from 3 mice each) or 2 ppm O_3 (3 pools from 3 mice each) and analyzed using an Agilent Bioanalyzer Small RNA Chip. (A) Plot depicting small RNA concentration data for each set of pooled EV samples. (B) A representative electropherogram from one pooled sample. Tags represent areas under the curve calculated between the vertical dashed lines for each peak.

Supplemental Figure 5. EV Small RNA Read Statistics. EV small RNA sequencing reads were trimmed of adapters, and aligned to the mouse reference genome (assembly NCBI37/mm9) using the miRquant 2.0 pipeline. (A) Plot depicting the percent of small RNA-seq reads at each step of the analysis pipeline. (B) Plot depicting the mean read lengths for all small RNAs mapped to the genome. (C) Pie-charts indicating the percentage of each small RNA species by exposure group.

Supplemental Figure 6. O_3-induced EV miR-2137 isoMir expression. Mature miR-2137 (solid border) and its isoMirs (dashed border) across each exposure group.

Supplemental Figure 7. Results of miRNA target enrichment analysis using miRhub. Plots depict the output of the miRhub algorithm for all lists used (one plot per list). Lists included all differentially expressed genes (All DE), downregulated genes (Down), upregulated genes (Up), or genes exhibiting up or down linear trends (Downtrend or Uptrend) for airway macrophages (dashed border) or conducting airway tissue (solid border). Lists included genes with a fold change of ± 2, and unadjusted p value < 0.05. Data points are plotted as $-\log_{10}(p\text{-value})$

generated by miRhub for each miRNA family, and points are highlighted if a member of the miRNA family was found to be differentially expressed in airway EVs.

Supplemental Figure 8. qRT-PCR validation of miR-22-3p expression. Taqman assays were used to determine the expression of miR-22-3p in EVs from mice exposed to 2 ppm O_3 versus FA in an independent experiment (FA, n=5; O_3, n=6). *$p<0.05$. Data were normalized to miR-23a-3p, a miRNA whose expression did not change with O_3, and presented as relative quantitative values compared to FA. Horizontal line denotes RQV of 1.

Supplemental Figure 1.

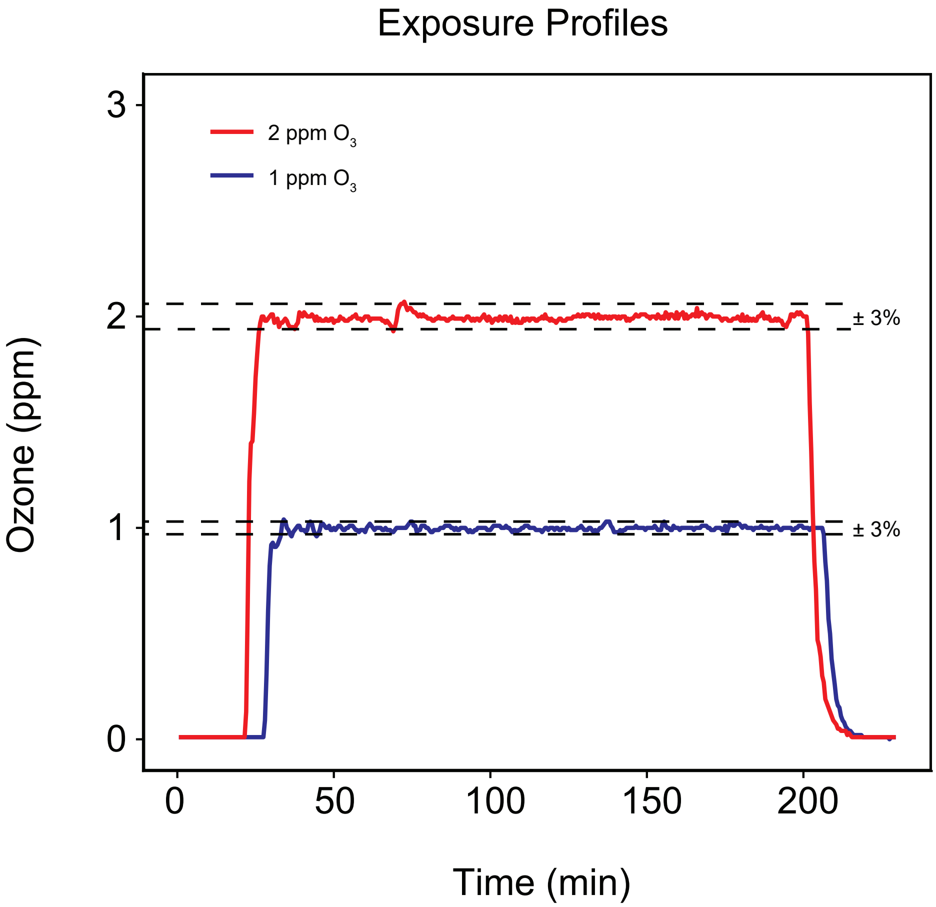

Supplemental Figure 2.

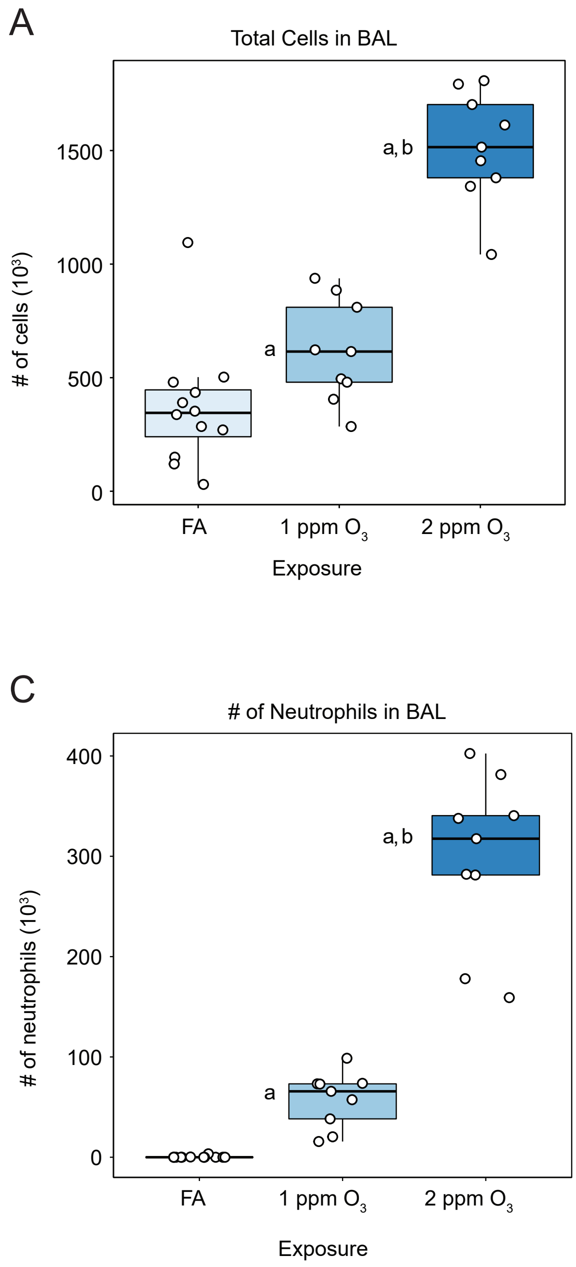

Supplemental Figure 3.

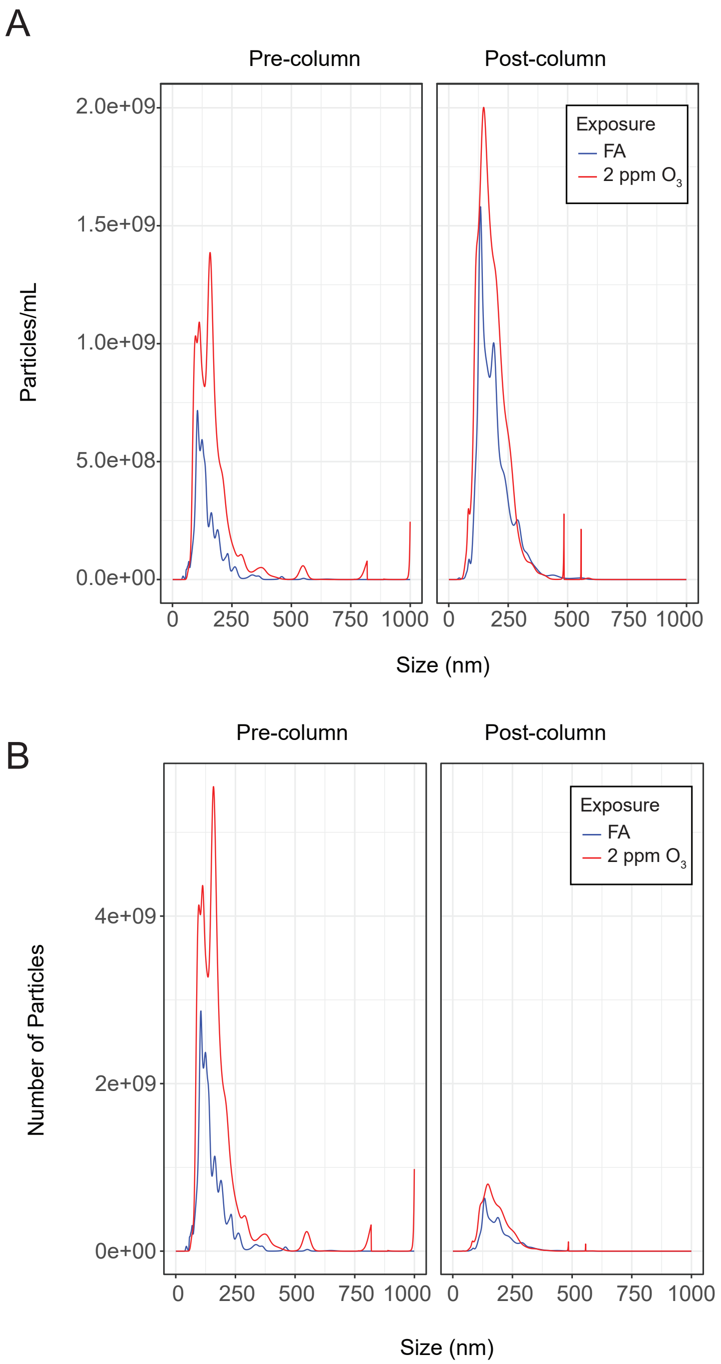

Supplemental Figure 4.

A

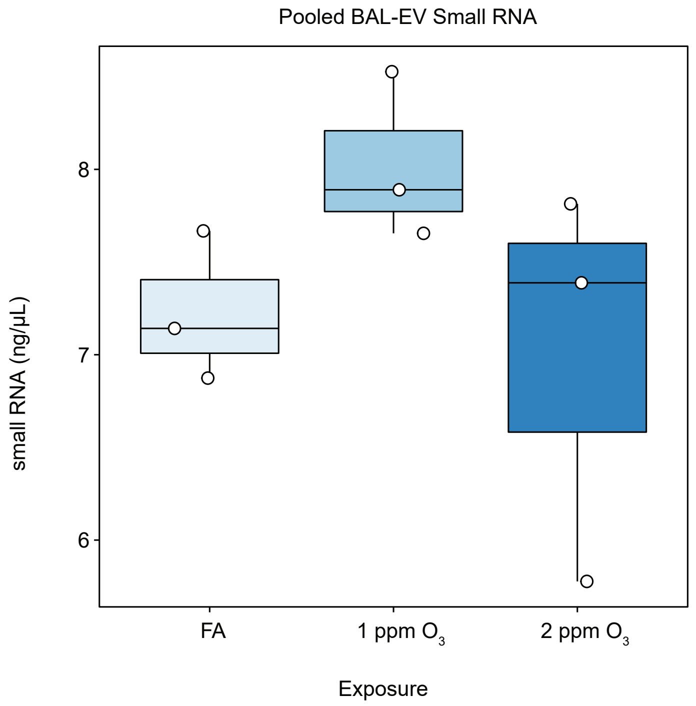

B

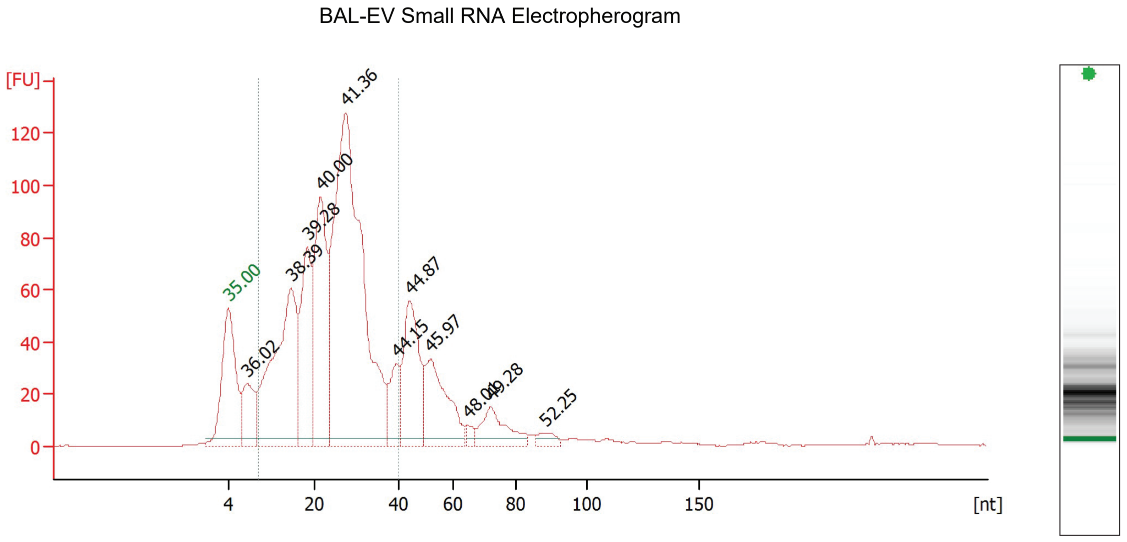

Supplemental Figure 5.

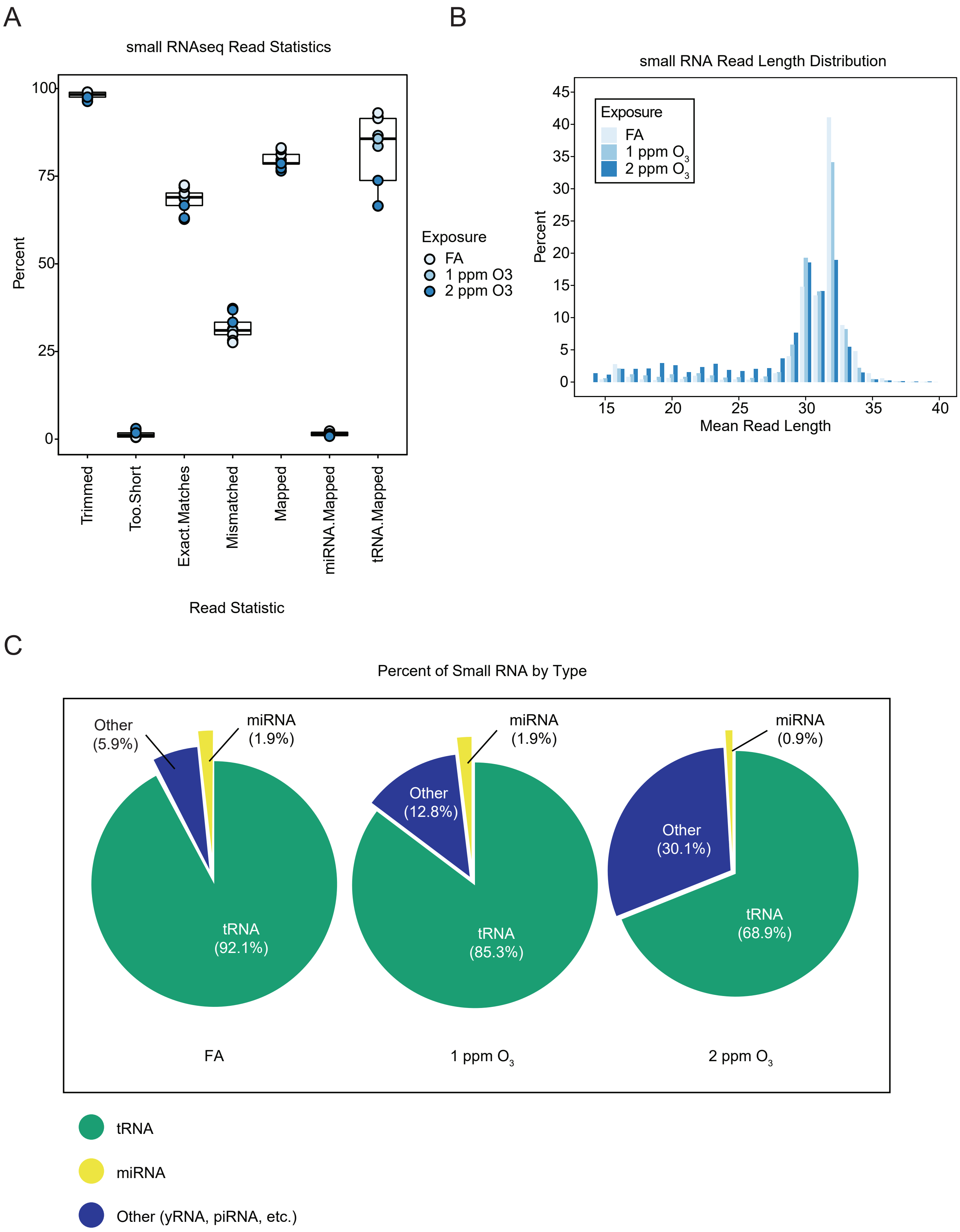

Supplemental Figure 6.

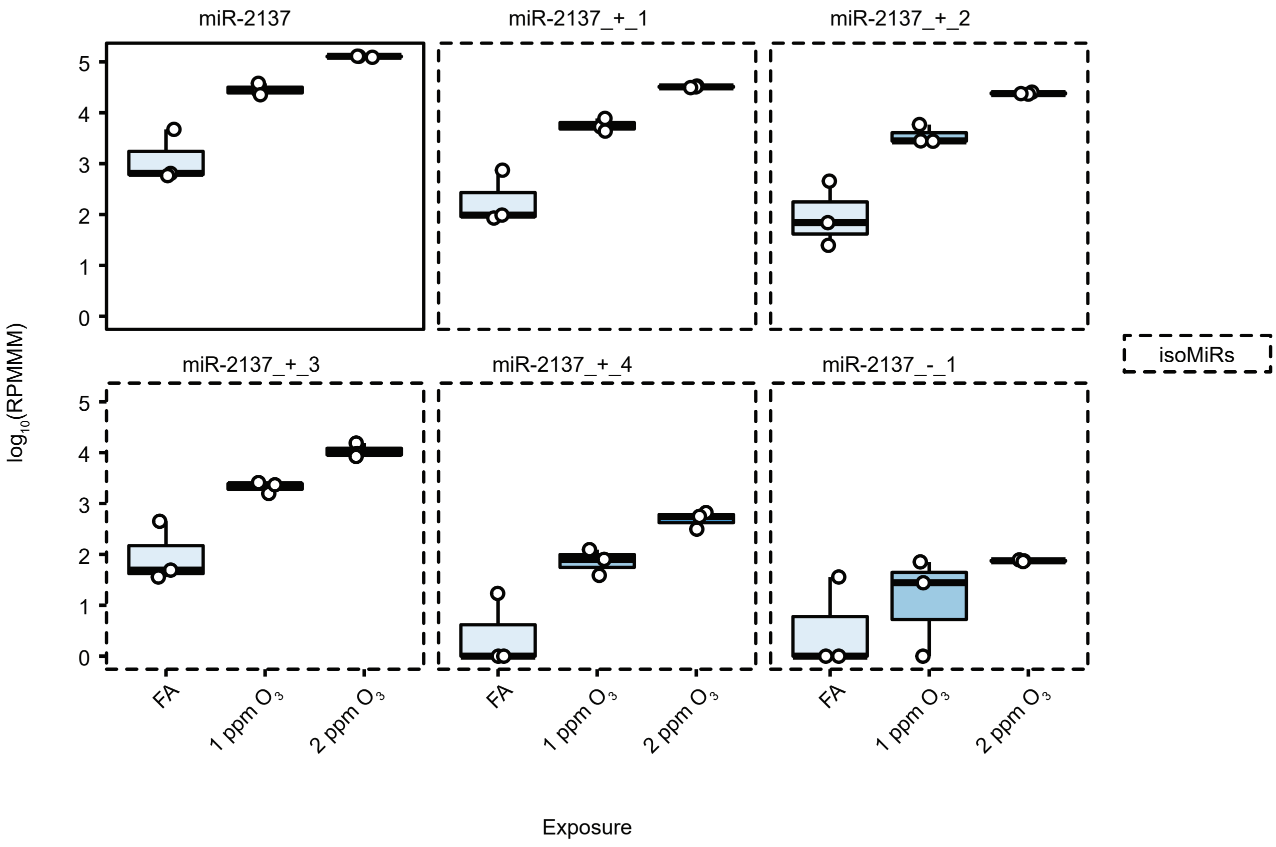

Supplemental Figure 7.

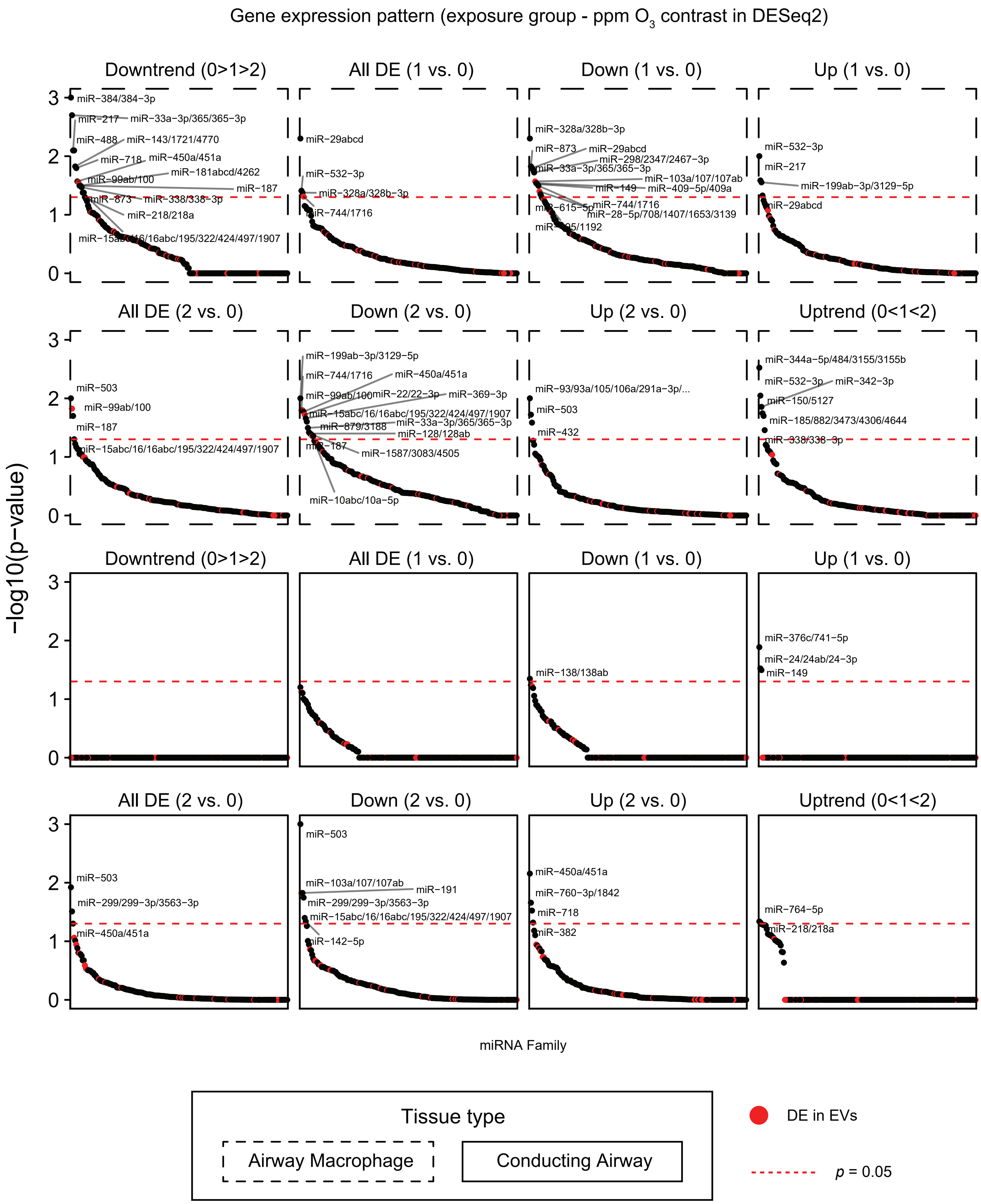

Supplemental Figure 8.

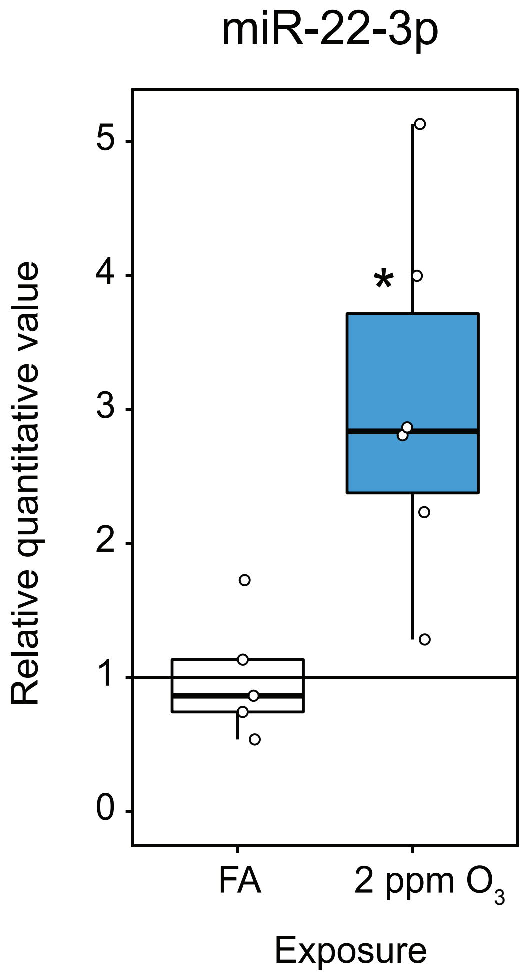

Supplement: Supplementary file 1 — Fig S1‐S8 [file PHY2-9-e15054-s001.pdf]
