# Supplementary material for: Ozone‐induced changes in the murine lung extracellular vesicle small RNA landscape
Source: Physiol Rep. 2021 Sep 23;9(18):e15054. doi: 10.14814/phy2.15054 (PMC8461034; doi:10.14814/phy2.15054)
Supplement: Supplementary file 2 — Supplementary Material [file PHY2-9-e15054-s002.docx]

**Ozone-induced changes in the murine lung extracellular vesicle small RNA landscape**

Gregory J. Smith, ^1^ Adelaide Tovar, ^1^ Matt Kanke, ^2^ Yong Wang, ^3^ Jessy S. DeShane, ^3^ Praveen Sethupathy, ^2^ and Samir N. P. Kelada ^2^

^1^Department of Genetics, University of North Carolina at Chapel Hill, Chapel Hill, North Carolina, USA; ^2^Department of Biomedical Sciences, College of Veterinary Medicine, Cornell University, Ithaca, New York, USA. ^3^Division of Pulmonary, Allergy, and Critical Care, Department of Medicine, School of Medicine, University of Alabama at Birmingham, Birmingham, Alabama, USA.

**Supplemental Table 1.** Exposure Characteristics

|  | Ozone (ppm) | | Relative Humidity (%) | | Temperature (C°) | |
| --- | --- | --- | --- | --- | --- | --- |
| Exposure | *Mean* | *SD* | *Mean* | *SD* | *Mean* | *SD* |
| FA-1 | < 0.01 | - | 42.57 | 1.10 | 24.93 | 0.36 |
| FA-2 | < 0.01 | - | 42.50 | 1.26 | 25.18 | 0.36 |
| 1 ppm O3 | 0.997 | 0.015 | 43.27 | 1.06 | 24.77 | 0.23 |
| 2 ppm O3 | 1.992 | 0.020 | 42.92 | 0.91 | 24.95 | 0.25 |
| Data are presented as arithmetic mean and standard deviation (SD) calculated from the time the concentration of O_3_ reached 90% of nominal until generation was stopped. | | | | | | |

**Supplemental Table 2.** Imaging flow cytometry analysis of eluted BAL extracellular vesicles

|  |  | | | |
| --- | --- | --- | --- | --- |
|  | FA | | 2 ppm O_3_ | |
| Antibodies | *Mean (%)* | *SD* | *Mean (%)* | *SD* |
| CD81 | 31.05 | 21.50 | 41.92 | 16.81 |
| SiglecF | 24.63 | 9.29 | 17.52 | 3.88 |
| CD51 | 22.27 | 4.74 | 19.25 | 2.37 |
| CD31 | 4.12 | 0.85 | 4.03 | 0.92 |
| CD11b | 3.61 | 0.95 | 4.50 | 1.63 |
| Ly6G* | 2.71 | 0.46 | 3.45* | 0.61 |
| EpCAM | 1.56 | 0.51 | 1.02 | 0.63 |
| CD9 | 1.53 | 0.58 | 1.77 | 1.05 |
| CD63 | 1.10 | 0.37 | 1.41 | 0.77 |
| CD81, CD51 | 4.13 | 1.18 | 4.36 | 1.02 |
| CD81, SiglecF | 3.49 | 0.95 | 2.78 | 0.49 |
| CD31, SiglecF | 3.37 | 1.74 | 1.73 | 1.50 |
| CD81, CD11b | 2.24 | 0.63 | 2.80 | 1.18 |
| CD51, SiglecF | 1.64 | 0.69 | 2.10 | 0.78 |
| CD51, CD31 | 1.54 | 0.31 | 1.36 | 0.26 |
| CD51, Ly6G | 1.51 | 0.17 | 1.73 | 0.45 |
| CD81, Ly6G* | 1.17 | 0.44 | 1.66* | 0.24 |
| Data represent percent (%) particles positive for each antibody combination and exposure. The arithmetic mean (%) and standard deviation (SD) are shown for each exposure group. N=6/group. *p <0.05, 2 ppm O3 vs FA. Antibodies positive in less than one percent of particles were excluded. | | | | |
